# Non-traditional therapies for diabetes: fact or fiction

**DOI:** 10.3402/jchimp.v2i2.18447

**Published:** 2012-07-16

**Authors:** Elena Forouhar, Paul Sack

**Affiliations:** 1 Elena Forouhar, Endocrine Fellow, MedStar Union Memorial Hospital, Baltimore, MD, USA; 2 Paul Sack, Assistant Chief of Endocrine Division, MedStar Union Memorial Hospital, Baltimore, MD, USA

**Keywords:** complementary and alternative medicine, Diabetes Mellitus, cinnamon, vinegar, fenugreek, ginseng, bitter melon, gymnema, chromium, vanadium

## Abstract

The number of medications now available to treat Type 2 Diabetes has been expanding quickly over the past two decades. At the same time, the use of complementary and alternative medicine (CAM) has also been rising. Individuals with diabetes are 1.6 times more likely than those without diabetes to use modalities that are not considered part of conventional medicine. Numerous dietary supplements are available over the counter and are being advertized to treat diabetes and its co morbidities. No conclusive data on their clinical benefit, potential harms, dosing or interaction with other medications is yet available. But for clinicians to maintain a trusting relationship with their patient, a respectful non-confrontational attitude is needed to encourage open dialogue, provide accurate information, and facilitate changes to the medical regimen. It is essential that clinicians stay informed and advise their patient with the available scientific data accordingly. In this review, we focus on current data on six supplements commonly encountered in community practice for treating diabetes, including cinnamon, fenugreek, vinegar, ginseng, bitter melon, gymnema, chromium, and vanadium.

## Introduction

There is an epidemic of Type 2 Diabetes Mellitus with a projected rise in worldwide prevalence from 171 million in 2000 to 366 million in 2030 (1). Complementary and alternative medicine (CAM) is also raising academic, industrial, and public interest as an option to be added to the constantly growing therapies for diabetes. The National Health and Nutrition Examination Survey found that 48% of diabetics use CAM (2). Diabetics are 1.6 times more likely to use CAM than non-diabetics (3). However, only 33% of patients using natural supplements or vitamins inform their health care provider (4). Despite lack of supporting medical evidence, use of herbal remedies has increased approximately 380% during the last 7 years in the United States (5). There are many reasons for this rise including traditional beliefs in different cultural or ethnic groups, lack of trust for the pharmaceutical industry, cost concerns, marketing and advertisement from the CAM companies, preference towards natural treatments, or the ease of access to information on the internet. Patients frequently perceive CAM as more trusted, with minimal side effects and lower cost.

In this brief review of available data, we aim to address the more common supplements that health care providers might encounter with their diabetic patients.

## What is complementary and alternative medicine (CAM)?

Complementary and alternative medicine is a type of treatment which is generally not taught in conventional medical school and is not currently an integral part of healthcare practices (3). Complementary medicine is defined as a treatment which is added to conventional medical practices, while alternative medicine replaces the established medical therapy. Common examples of CAM are acupuncture, hypnosis, functional food therapy, nutritional supplements, herbal remedies and traditional medicines like Chinese, Ayurvedic or Native American. While there are numerous options that are advertised to help diabetics, this article will focus on some of the most common nutritional supplements used for diabetes and highlight the best available scientific data regarding their use.

## Cinnamon

Cinnamon is a warm and sweet spice and aroma highly valued in cuisine, but is also used for multiple medical maladies including diabetes. *In vitro* studies on adipocytes show that an active component of cinnamon can potentiate insulin effects and improve glucose uptake (6). *In vivo*, it appears to enhance the insulin signaling pathway and improve insulin resistance. In one study on non-diabetic healthy subjects, adding cinnamon to rice pudding did not affect glucose level (7), but other studies in diabetics showed some improvement with cinnamon supplementation. In one such study, Type 2 diabetics with HbA1C>7 supplemented with 1 g of cinnamon per day had a reduction in HbA1C of 0.8% (8). In another study, cinnamon supplementation in a group of diabetics improved mean fasting glucose by 18–29%, triglycerides by 23–30%, LDL by 7–27%, and total Cholesterol by 12–26% (9). However, a Meta analysis on five trials with 282 subjects failed to show any benefit of cinnamon supplementation on HbA1C, fasting glucose or lipids (10). Hence, while there is biological plausibility that cinnamon can affect insulin mediated glucose uptake, insulin sensitivity and possibly GLP-1 secretion, current data is conflicting and no dose recommendation is available. While more trials are needed, there is no reason to discourage patients from taking cinnamon supplements or adding some extra to their food.

## Vinegar

Vinegar has been used for thousands of years for its excellent taste in cuisine. It has many reported medicinal uses including lowering cholesterol, improving diabetes, and increasing satiety. Vinegar has been shown to attenuate the serum glucose and insulin responses to a load of sucrose or starch (11). Johnson and colleagues studied Type 2 diabetics with an average HbA1C of 6.7. In test group, 2 ounces of apple cider vinegar was added to the standard meal of a bagel, butter and orange juice. The fasting glucose levels improved by 4% in the diabetic group compared to 2% in the control group. Insulin sensitivity improved by 34% and 19% in insulin resistant and Type 2 diabetics respectively (12). There are multiple hypothesized mechanisms for these effects including a decreased glycemic index of food, decreased gastric emptying, decreased hepatic gluconeogenesis, improvement in satiety, and increased insulin sensitivity. Other studies on vinegar are suggestive of some improvement in fasting or even postprandial hyperglycemia (13–15) but large clinical trials with flawless methodology remain elusive. It certainly does not hurt to recommend adding taste to food with vinegar while waiting for more convincing evidence.

## Fenugreek

Fenugreek is a plant of Mediterranean origin first used in ancient Egyptian cuisine. It also has medicinal usages in Ayurvedic and Chinese medicine. The active component is an amino acid, 4-hydroxyisoleucine, which is isolated from the seeds. Animal data, including both rats and dogs, show remarkable improvement in glucose and insulin increment after IV glucose load in the group who received IV 4-hydroxyisoleucine (16). However, clinical studies are limited. One study done in Iran, on 24 Type 2 diabetics showed 10 g a day of Fenugreek for 8 weeks decreased fasting blood glucose from 182 to 136 and decreased triglycerides from 245 to 172, but did not change the HbA1C (17). A study on 160 Type 2 diabetics in Jordan showed a dose dependent improvement in 2 hour post prandial sugars in the group receiving 5 g Fenugreek compare to 2.5 g and the control group (18). In another study on healthy subjects, Shakib et al. found those who consumed bread with fenugreek had an improvement in the postprandial glucose response compared to those who ate bread without fenugreek (19). These changes may be due to a decrease in gastric emptying, decrease carbohydrate absorption, improvement in glycemic index, or through an insulinotropic or secretagogue effect (16). More clinical trials are needed to investigate its long term benefits. It can also have an anti-platelet effect. Therefore, caution is advised for those patients who are on anticoagulants.

## Ginseng

Ginseng is one of the Chinese traditional herbs widely used for thousands of years in cooking, but also as a tonic and restorative agent which has been purported to increase vitality, mental and physical performance, and body metabolism. Limited clinical data has suggested that the berry extract of plant has a blood glucose lowering effect. In obese, diabetic mice (ob/ob mice) intraperitoneal ginseng berry extract decreased fasting blood glucose (20). In one human study on 10 non-diabetic and 9 diabetic subjects, 3 g of ginseng given 40 min before a 25 g glucose challenge attenuated the post prandial glucose excursions in both diabetics and non-diabetics (5). In another study, 8 weeks of supplementation with ginseng in 36 Type 2 diabetics showed a reduction in fasting blood glucose and a decrease in HbA1C by 0.5%. The mechanism for this effect is unknown. It may be due to delayed gastric emptying or a decrease in carbohydrate absorption. An even more interesting hypothesis is that there is a nitric oxide (NO) mediated increase in glucose transporter protein as shown in the mice liver and sheep erythrocytes (21, 22). It was recently shown that insulin-stimulated glucose uptake in rat skeletal muscles and adipose tissue is NO dependent. Enhanced NO synthesis by ginseng in the endothelium of lung, heart, and kidney as well as in the corpus cavernosum has already been reported (23). Larger studies are needed to validate ginseng's therapeutic benefit and its mechanism in diabetes.

## Bitter melon (Momordica Charantia)

Bitter melon is a light green fruit, popular for thousands of years in Asian and Indian cuisine. People from many different cultures use bitter melon for treating diabetes. Abundant pre-clinical studies support its anti-diabetic and hypoglycemic effects. In animal studies, all parts of the plant showed a hypoglycemic effect (24). Some studies compared its effect to oral medications like sulfonylureas. There are several potential mechanisms proposed including activation of the AMP-activated protein kinase (AMPK) in insulin signaling cascade, activation of peroxisome proliferator-activated receptors (PPAR-gamma and alpha), and an increase in intracellular glucose transport by translocating glucose transporter type 4 (25). One of the proteins isolated from Bitter Melon showed insulin mimetic properties (26). The animal and *in vitro* data on Bitter Melon is interesting but clinical studies are sparse, sporadic, inconclusive and heterogeneous. Better-designed clinical trials with sufficient sample size are needed to vindicate the acclaimed efficacy of Bitter Melon.

## Gymnema

Gymnema is an Ayurvedic herb used for nearly 2000 years. Gymnema reduces the taste of sugar in the mouth and can potentially help with sugar cravings. *In vitro* studies on isolated mouse and human β-cells, showed a reversible increase in intracellular calcium and insulin secretion by extracts of Gymnema. It may also decrease liver glucose production as well as help with regeneration of the cells in the pancreas (27–30). Limited clinical trials have been promising but large-scale clinical trials are needed to evaluate its safety and efficacy.

## Chromium

Chromium is an essential trace metal which is also known as ‘glucose tolerance factor’ by potentiating the action of insulin (31). *In vitro* studies demonstrate that chromium binds to the oligopeptide chromodulin inside the cell to form chromium-chromodulin complex which upregulates the tyrosine kinase activity and inhibits phosphotyrosine phosphatase activity at the insulin receptor level, an early point in the signaling cascade. This results in amplification of the insulin signaling (32).

Chromium is naturally available in brewer's yeast. It is also manufactured as biologically active formulations like chromium picolinate. Manufacturers promote the benefits of chromium in the prevention and treatment of insulin resistance, Type 2 diabetes, dyslipidemia, or cardiovascular disease. However, the effect of chromium supplementation in individuals who are not severely chromium deficient is unclear (32). In old case reports, infusing chromium to critically ill patients with severe insulin resistance has improved their insulin requirements significantly (32).

A systematic review of the effects of chromium supplementation on glucose metabolism and lipid levels included 1198 participants with diabetes, impaired glucose tolerance, or no diabetes who were placed on different formulations of chromium or brewer's yeast (33). The overall analysis showed no statistically significant improvement in HBA1C, fasting glucose, post prandial glucose, insulin sensitivity index, or lipoprotein levels. In some studies, chromium supplementation in patients with Type 2 diabetes had a mild to modest beneficial effect on glucose and lipid level. However, the studies with the poorest quality showed the greatest effect on HbA1C lowering, making the reported benefit suspect. In some studies, brewer's yeast supplementation, but not chromium picolinate supplementation, raised HDL cholesterol. The overall poor quality across these studies limits the strength of these conclusions (34).

## Vanadium

Vanadium is a metal which is not essential for survival. Clinical deficiency states have never been reported but some insulin-mimetic action is ascribed to it. *In vitro* and in animal studies pharmacologic doses of vanadium can mimic almost any known bioeffect of insulin through an insulin receptor independent pathway. It enhances protein kinase activity, inhibits phosphotyrosine phosphatase, enhances glucose transport and glycogenesis, and inhibits ketogenesis and lipolysis (35). A few clinical studies on diabetic patients showed that small doses of vanadium over a three-week period improved glucose and lipid metabolism, glucose utilization, and insulin sensitivity (35). However, enough clinical research is not yet available. The toxicity of these compounds is also of concern. Excess vanadium can result in severe diarrhea, hepatotoxicity, and teratogenicity (36). The routine use of vanadium as a pharmacologic treatment for diabetes cannot yet be recommended.

## Conclusion

There are numerous herbal remedies and nutritional supplements available to our diabetic patients. While there is some limited data as their benefit, none have reached the level of evidence that US Food and Drug Administration-approved medications require. In addition, there is minimal regulation on these treatments. There is no burden of proof to show benefit or prove safety. The only time a regulatory agency gets involved is if there is inappropriate advertising or enough concern for side effects. However, our patients will continue to use CAM and it is important to not be judgmental or confrontational. This will help to establish a trust so that patients will feel free to share what alternative therapies they are using.

There are some practical issues to consider when patients are using CAM. The cost of these therapies may make it difficult for the patient to afford their prescribed medications. It is also essential to emphasize that none of these treatments are approved as a substitute for their prescription medications. If there is a therapy that is unfamiliar to the physician, it is important to, at least briefly, become familiar with it to be sure that there are no major known side effects or drug interactions.
